# Spatial Distribution of COVID‐19 Diagnostic Services in Mopani District, Limpopo Province, South Africa

**DOI:** 10.1002/puh2.70161

**Published:** 2025-10-28

**Authors:** Kuhlula Maluleke, Alfred Musekiwa, David Mckelly, Ethel Baloyi, Tivani Mashamba‐Thompson

**Affiliations:** ^1^ School of Health Systems and Public Health Faculty of Health Sciences University of Pretoria Pretoria South Africa; ^2^ Inclusive Smart Settlements and Regions (ISSR) Smart Places CSIR Stellenbosch Central Stellenbosch South Africa; ^3^ Legisature Department Ekurhuleni Metropolitan Municipality Germiston South Africa

## Abstract

**Introduction:**

Access to healthcare facilities in rural areas remains a major challenge, particularly during the coronavirus disease 2019 (COVID‐19) pandemic. This study investigated the spatial distribution and accessibility of COVID‐19 point‐of‐care (POC) diagnostic services in Mopani District, Limpopo province, South Africa, using a geographic information system (GIS)‐based approach. The aim was to identify areas where healthcare access requires improvement.

**Methods:**

A descriptive cross‐sectional study design was used, analysing secondary data through dasymetric mapping to disaggregate and re‐aggregate population data into analytical units. Accessibility was measured by distances from residences to the nearest primary healthcare (PHC) clinics (<5 km) and from clinics to the nearest district hospitals (<30 km). Demographic and socio‐economic data from Statistics South Africa were included for context.

**Results:**

Mopani District had an estimated population of 1,202,916, with 942,801 (78.4%) residing within 5 km of a PHC clinic. The district had 105 clinics, each serving about 11,456 people, and 72 (68.6%) of these clinics were within 30 km of a district hospital. The district contained both densely and sparsely populated areas, with high unemployment and low‐income levels, particularly in rural regions. Limited public transport further constrained access. Ba‐Phalaborwa, Maruleng, Greater Tzaneen and Greater Letaba have relatively good access, whereas Greater Giyani faces significant challenges.

**Discussion:**

Although a majority of the population lived within 5 km of a clinic, notable gaps in accessibility remain. Improving transport infrastructure, using telemedicine and mobile health units, and implementing socio‐economic support strategies, such as subsidized transport, can enhance access. Addressing geographic and structural inequalities in healthcare distribution is critical to promoting equity and improving health outcomes in rural districts like Mopani.

## Graphical abstract

This study maps diagnostic service accessibility in Mopani District, South Africa, revealing gaps in rural healthcare coverage. Travel distances to primary healthcare clinics and district hospitals highlight areas needing improved infrastructure and targeted interventions to enhance equitable access to essential diagnostic services.

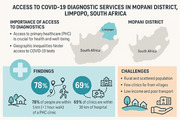

## 1 Introduction

Access to primary healthcare (PHC) is a fundamental human right [1]. Governments worldwide strive to ensure that all members of society have equal and adequate access to PHC, regardless of socio‐economic and geographic factors. Adequate access to PHC is also critical for achieving Sustainable Development Goal (SDG) 3, which aims to ensure good health and well‐being for all [2].

The coronavirus disease 2019 (COVID‐19) pandemic severely tested public health systems, particularly in low‐ and middle‐income countries (LMICs) where insufficient resources, inadequate infrastructure and poor access to health services exacerbated disease outcomes [3]. In response, the World Health Organization (WHO) recommended scaling up COVID‐19 point‐of‐care (POC) diagnostic testing in resource‐limited settings with poor access to laboratory infrastructure [4, 5]. The access to COVID‐19 tools (ACT) accelerator, a global collaboration to accelerate the development, production and equitable access to COVID‐19 tests, has supported high‐burden countries and areas with the greatest need, including resource‐limited settings in South Africa [6].

South Africa's health sector faces significant challenges in developing a unified national health system capable of delivering quality healthcare to all citizens [7]. There are large disparities in the spatial distribution of health services, with facilities often located in fixed locations, whereas health needs vary across space and time [8, 9, 10]. This issue is particularly pronounced in resource‐limited settings where the most immediate factor affecting geographical accessibility is the distance and time required to travel to well‐equipped and adequately staffed healthcare facilities [11]. Studies in LMICs have consistently shown that physical proximity to healthcare services plays a crucial role in their utilization [12]. Healthcare facilities are usually situated in densely populated areas, leaving people in remote settings to travel longer distances to access PHC [11]. When distance to healthcare hinders accessibility, the detection of infectious diseases may be delayed, causing mild infections to progress to severe disease and potentially leading to suboptimal care or even mortality [13, 14, 15]. In this study, geographical accessibility was defined as the physical distance travelled to the nearest healthcare facility.

Recent studies have advanced methodological approaches for assessing healthcare accessibility. For example, Krishnakumari et al. [16] assessed geographical accessibility to COVID‐19 testing facilities in Nepal using high‐resolution population grids and modelled travel time rather than straight‐line distance. Similarly, Kalonde et al. [17] applied travel‐time‐based modelling to define health facility catchment areas in Blantyre, Malawi and validated these modelled catchments against patient origin data, providing a benchmark for empirically grounded accessibility modelling. These studies demonstrate the value of travel‐time surfaces and patient‐derived validation for enhancing the realism of accessibility analysis. In contrast, the present study focuses on network‐based distance as a proxy for accessibility, a pragmatic choice given data availability in Mopani District, South Africa. By situating our analysis alongside these methodological advances, we highlight both the contribution of distance‐based modelling in resource‐limited settings and opportunities for future refinement through travel‐time approaches and catchment validation.

Understanding the regional progression and impact of COVID‐19 in South Africa was crucial for assessing the pandemic's management and outcomes. According to the NICD's definition, a COVID‐19 wave was marked from when weekly cases reach 30 per 100,000 people until they drop below this threshold [18]. Provincial timelines varied significantly. For instance, in Limpopo, the first wave spanned from 16 March to 31 October 2020, and the second wave lasted from 1 November 2020 to 31 March 2021. During these periods, Limpopo had a COVID‐19 inpatient case fatality rate of 27.1%, higher than the national average of 25.8% [19]. In the Mopani District within Limpopo, the first wave saw 12,545 cases and 301 deaths, whereas the second wave recorded 59,662 cases and 1960 deaths, resulting in a case fatality rate of 27.5% [20].

This study investigates the spatial distribution of COVID‐19 diagnostic services in relation to population density in the Mopani District, Limpopo Province, South Africa. Providing health facilities to all settlements in the district is challenging due to the varying sizes and scattered nature of most settlements. To fully understand the spatial distribution of COVID‐19 diagnostic services in relation to population density, we identified the physical locations of PHC clinics and district hospitals (areas of supply), mapped the population distribution (areas of demand), calculated the travel distances from areas of demand to areas of supply and examined variations in spatial accessibility across different areas. Finally, we identified areas with a paucity of COVID‐19 diagnostic services, conducting a geographic information systems (GIS)‐based accessibility analysis to achieve this. Despite the critical importance of accessible healthcare services, especially during a pandemic, there is limited research focusing on how the distribution of such services aligns with population density in rural areas. By investigating this relationship, we aim to provide valuable insights into the challenges and opportunities for optimizing healthcare accessibility, which can inform policy decisions and resource allocation strategies in similar settings.

## 2 Materials and Methods

### 2.1 Study Design

In this descriptive cross‐sectional study, a GIS approach was used to investigate the spatial distribution of COVID‐19 diagnostic services. This study design was useful for collecting benchmark data that demonstrate the size of a problem (distance travelled to access COVID‐19 diagnostic services) at a specified point in time [21].

### 2.2 Study Setting

**FIGURE 1:**
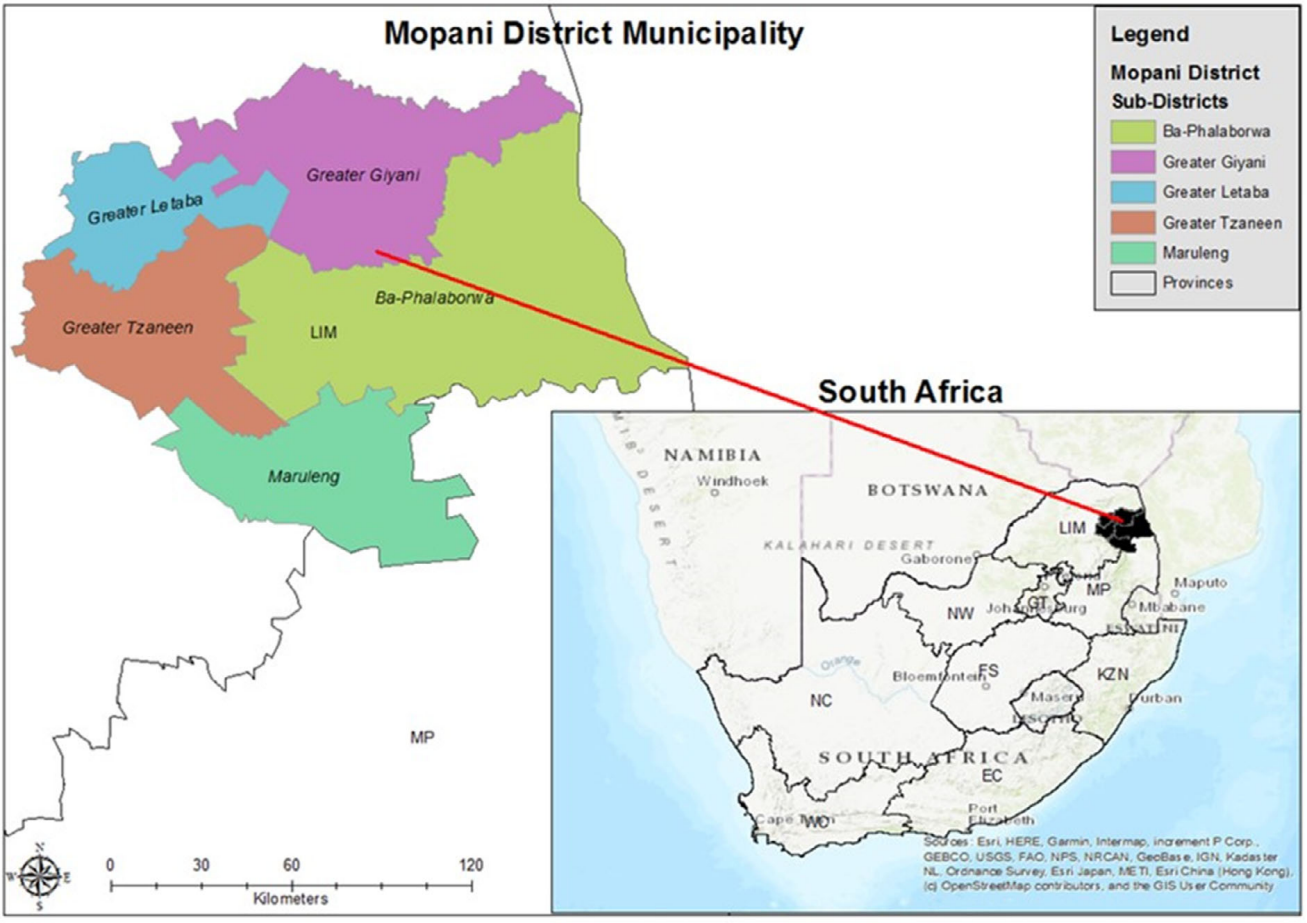
Location of the Mopani District, Limpopo Province, South Africa.

The study was set in Mopani District located in the Limpopo Province in South Africa (Figure 1). In 2021, a total of 1,207,020 people lived in the province (20,011 km^2^) [22]. In the Mopani District, 81% of people lived in rural areas, 14% lived in urban areas, whereas 5% lived on farms [23]. Mopani District consists of five sub‐districts, namely, Greater Giyani, Greater Letaba, Greater Tzaneen, Ba‐Phalaborwa and Maruleng (Figure 1). Most of the population relied on the public health system for essential diagnostic services.

### 2.3 GIS Accessibility Analysis

ArcGIS 10.8.2 software was employed to determine (i) travel distances from places of residence to the nearest PHC clinics and (ii) travel distances from PHC clinics to the nearest district hospital where National Health Laboratory Services (NHLS) were located. The GIS accessibility analysis followed a stepwise approach similar to that described in Baloyi et al. [24], focusing on PHC clinics. Initially, the study area was divided into finer analysis units, and the population was allocated to these units. The next step incorporated the road network and the locations of PHC clinics and district hospitals to conduct a travel distance analysis, identifying areas with adequate and inadequate access to COVID‐19 diagnostic services and analysing spatial variations by sub‐district. Finally, the results were visualized through travel distance maps, a bubble map showing the population within 5 km of clinics, and a map indicating the distance from clinics to the nearest hospital.

### 2.4 Data Manipulation

To investigate spatial distribution of COVID‐19 diagnostic services in Mopani District, three main data types were used: (i) the population distribution, (ii) the physical location of the PHC clinic and (iii) the spatial layout of the road network.

### 2.5 Physical Location of PHC Clinics and District Hospitals (Areas of Supply)

**FIGURE 2:**
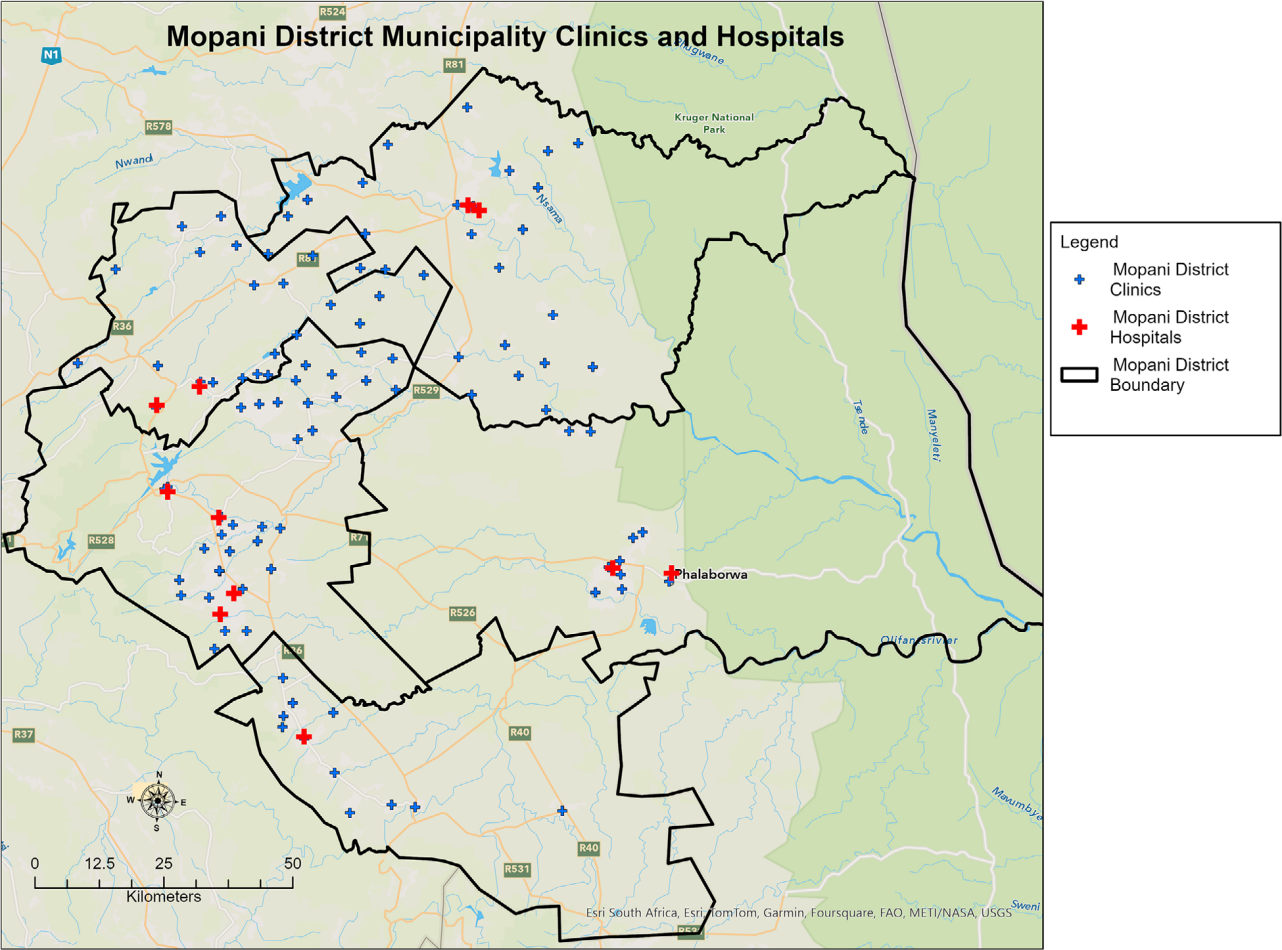
Location of PHC clinics and district hospitals in Mopani District, Limpopo province, South Africa.

The district had 105 PHC clinics and seven district hospitals [25]. There were 28 PHC clinics in Greater Giyani, 10 in Ba‐Phalaborwa, 11 in Maruleng, 35 in Greater Tzaneen and 21 in Greater Letaba (Figure 2). All the districts had one hospital, except for Greater Tzaneen and Greater Letaba that had two district hospitals each. All the included PHC clinics offered COVID‐19 POC diagnostic services, were administered by the public sector and had a fixed geographical location. The geographic coordinates of the PHC clinics were obtained from the South African National Department of Health (NDoH) website (https://www.health.gov.za/) and the Mopani District Municipality GIS unit [25]. The two datasets were merged, and duplicate coordinates for each PHC clinic were removed. Due to the lack of spatially precise residential data, the PHC clinic's catchment areas were used to ascertain the residences of the patients.

### 2.6 Population Disaggregation (Areas of Demand)

**FIGURE 3:**
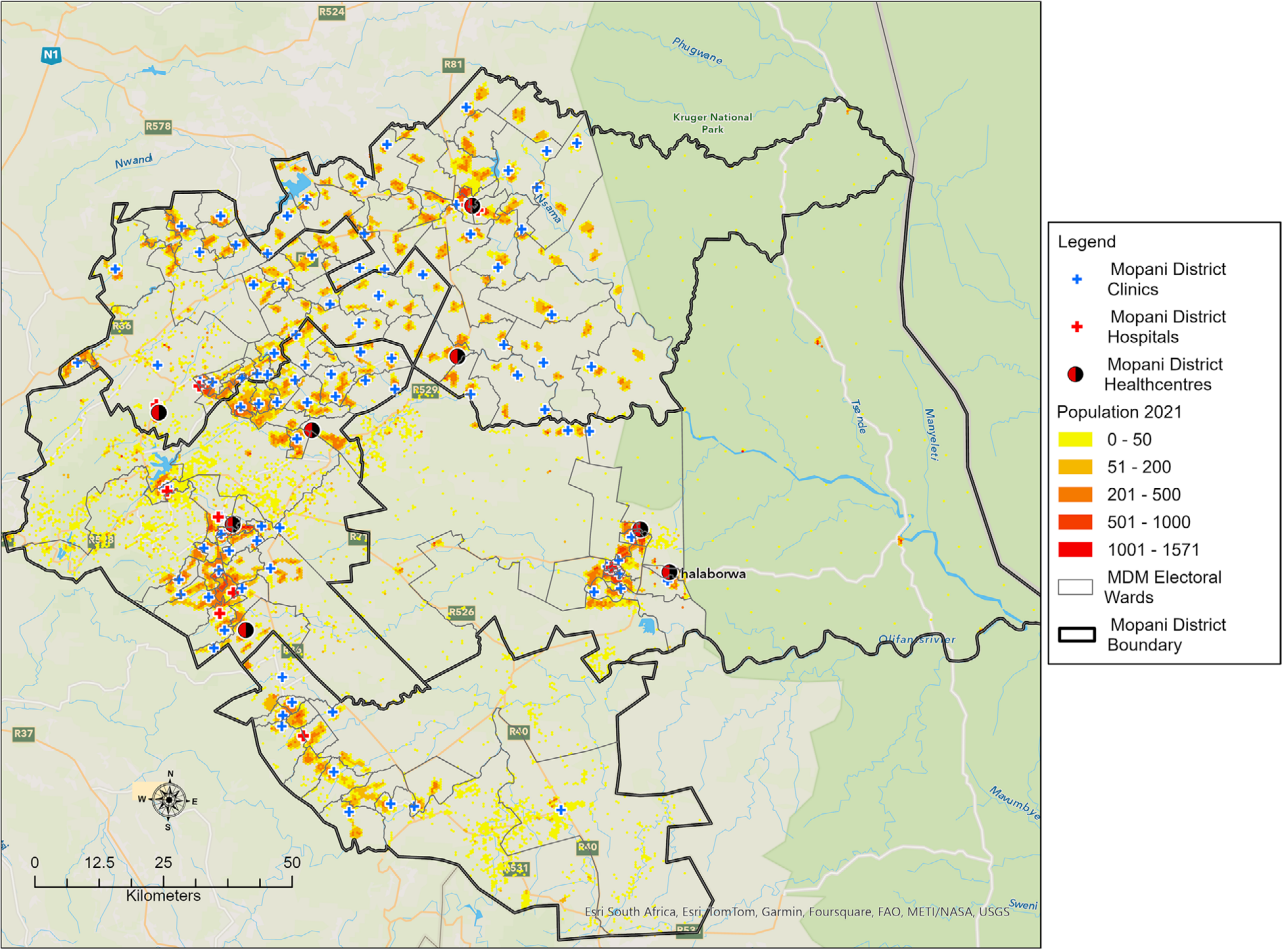
Population distribution of the Mopani District in 2021.

The density‐weighted dasymetric mapping technique used by the authors involved dividing the Mopani District Municipality into 20‐ha hexagons and using ancillary data, such as land‐use information, to assign population density weights to different areas. The total population was then redistributed across the hexagons on the basis of these weights, ensuring that areas with higher density weights receive a proportionally larger share of the population. This method provided a precise representation of population distribution by reflecting actual spatial patterns and densities [26]. The 2021 community survey conducted by Statistics South Africa (https://www.statssa.gov.za/) formed the base data for the analysis [22]. Figure 3 shows the 2021 population of the Mopani District on a dasymetric map.

### 2.7 Road Network Data

**FIGURE 4:**
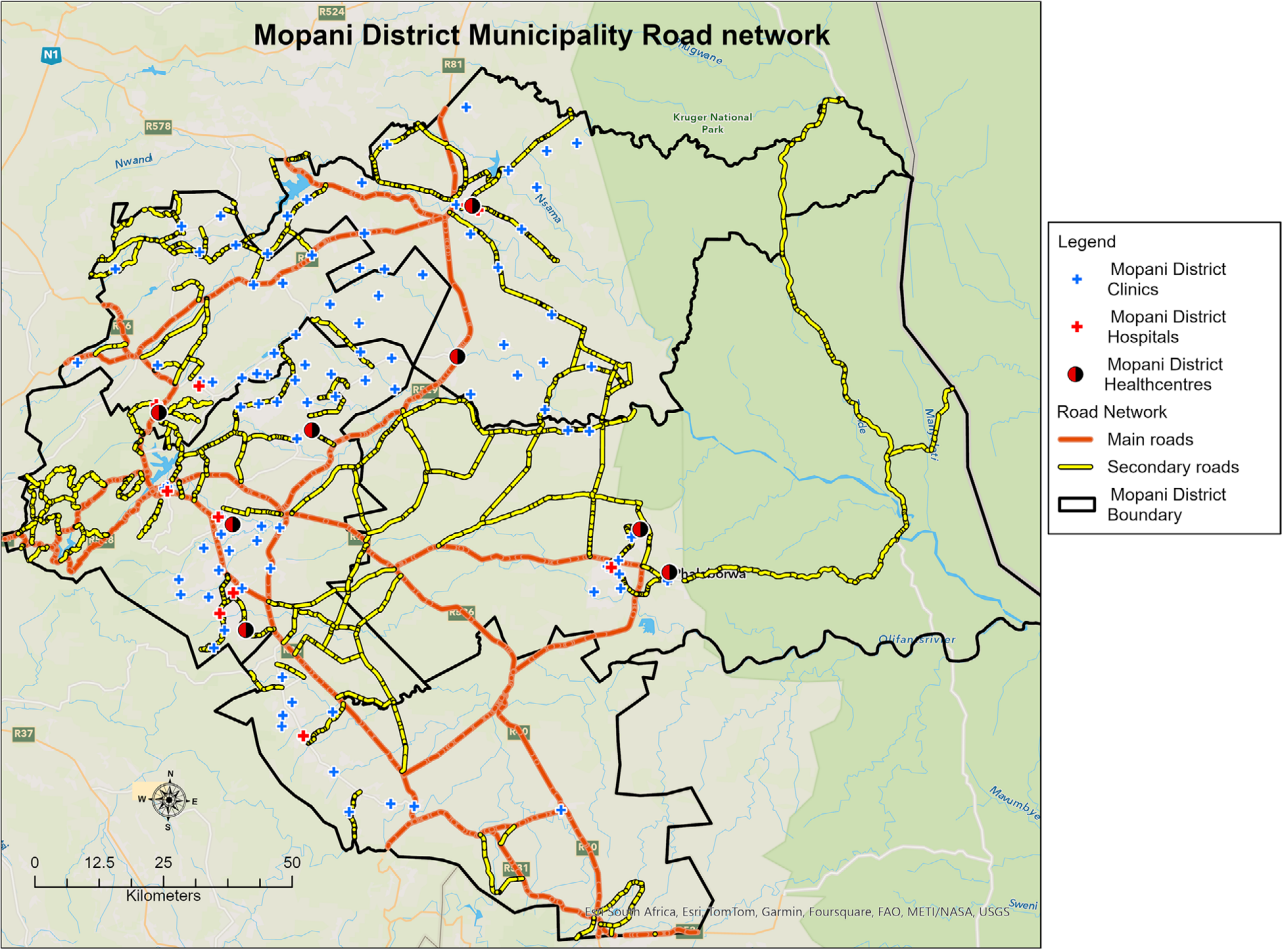
Mopani District road network used for the travel distance analysis.

Geographic accessibility was measured using the road network distance, which was more realistic than straight line distance. In this study, accessibility was modelled using network‐based travel distance (in kilometres) rather than estimated travel time. Although distance provides a meaningful measure of proximity, it does not fully capture variations introduced by topography, road conditions or transport availability. The road network distance was measured on a flow map from each hexagon centroid to the PHC clinic using an origin–destination matrix. A road network for the Mopani District was obtained from the GIS unit at Mopani District Municipality (Figure 4). The implicit mode of travel was used to calculate distance between locations. In rural areas where the implicit mode of travel is walking or public transport, accessibility analyses often assume that individuals travel primarily by foot or rely on available public transportation options, such as taxis and buses. This assumption shapes the way distances to healthcare facilities are calculated, typically favouring routes that are walkable or covered by public transport [27]. Consequently, the analysis may highlight the need for improved transportation infrastructure and closer healthcare facilities to better serve the population's needs.

### 2.8 Input Standards

It was assumed that patients would travel to the nearest PHC clinic for COVID‐19 diagnostic services. We categorized people who lived within <5 km as having high accessibility; 5–10 km, moderate accessibility; and >10 km, poor accessibility based on the CSIR guidelines for the provision of social facilities in South Africa [28]. The ideal distance from a PHC clinic to the nearest district hospital was 30 km; therefore, any distance above this threshold was deemed inaccessible [28].

## 3 Results

Across the entire Mopani District, which encompassed a total population of 1,202,916 and an overall population density of 11,456 individuals per clinic, there were 105 clinics. On average, 78.4% (942,801 individuals) of the population resided within 5 km of a clinic, whereas 21.6% (260,115 individuals) were beyond this distance. Regarding proximity to district hospitals, 68.6% (72 clinics) were within 30 km, with 31.4% (33 clinics) being farther away (Table 1).

**TABLE 1:** Population data per sub‐district.

Sub‐district	Population	Number of clinics	Population density	Population within 5 km (%)	Population beyond 5 km (%)	Clinics within 30 km (%)	Clinics beyond 30 km (%)
Ba‐Phalaborwa	186,741	10	18,674	159,401 (85.4)	27,340 (14.6)	8 (80)	2 (20)
Greater Giyani	265,721	28	9490	159,339 (60)	106,382 (40)	15 (53.6)	13 (46.4)
Greater Letaba	215,478	21	10,261	168,292 (78.1)	47,186 (21.9)	10 (47.6)	11 (52.4)
Greater Tzaneen	431,957	35	12,342	382,071 (88.5)	49,886 (11,5)	29 (82.9)	6 (17.1)
Maruleng	103,019	11	9365	73,698 (71.5)	29,321 (28.5)	10 (90.9)	1 (9.1)
**Total**	**1,202,916**	**105**	**11,456**	**942,801 (78.4)**	**260,115 (21.6)**	**72 (68.6)**	**33 (31.4)**

**FIGURE 5:**
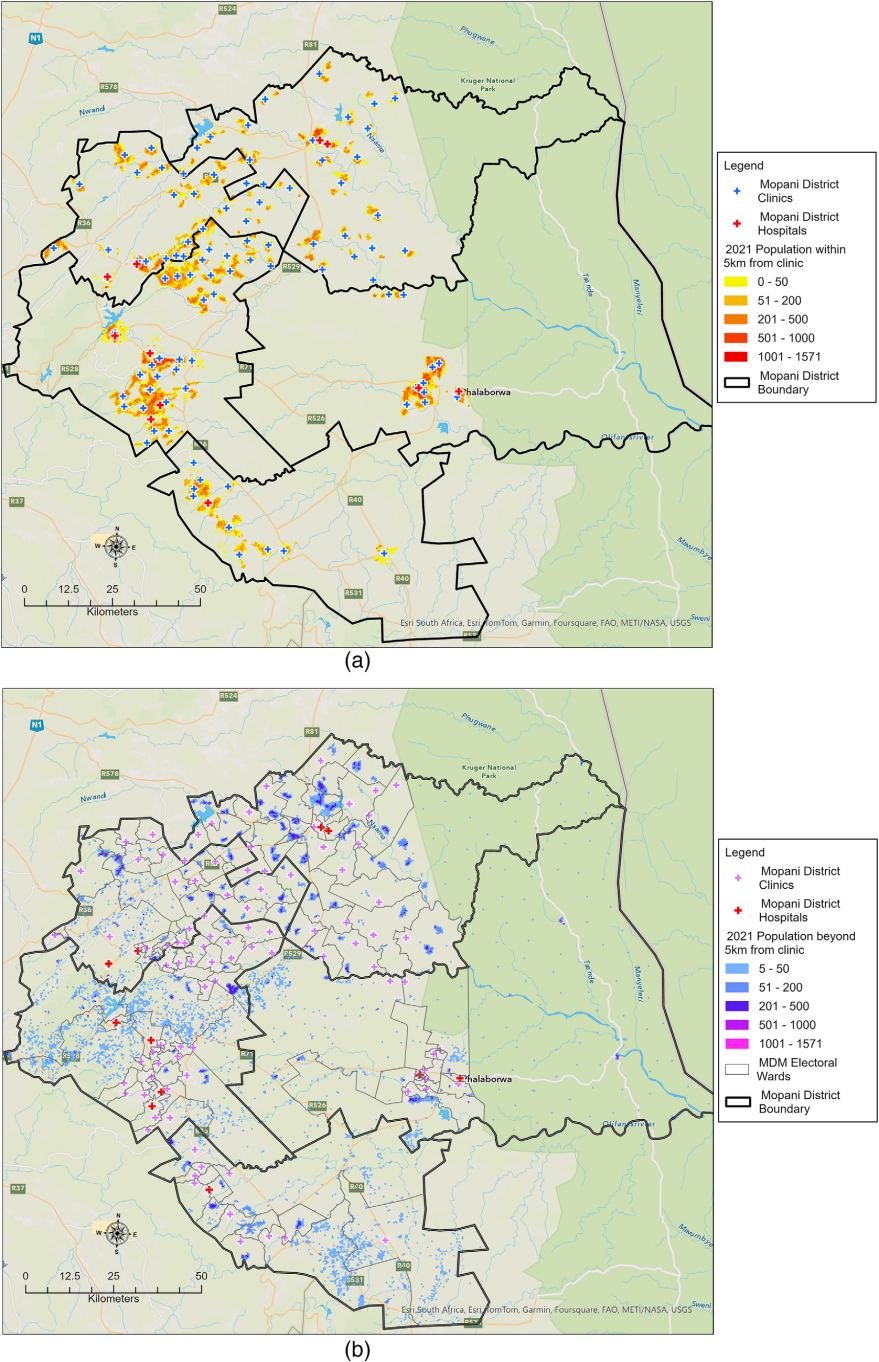
The travel distance analysis showing population density in relation to the location of public health clinics. The figures show population density within 5 km of a PHC clinic (a) and (b) more than 5 km from a PHC clinic.

Figure 5a displays the spatial distribution of the population in the Mopani District who were able to access PHC clinics providing COVID‐19 diagnostic services within a 5‐km travel distance, with a mean travelling distance of 2.95 km (95% CI: 2.46–3.45 km). The district's built‐up areas, which included major towns and their surroundings such as Phalaborwa, Giyani, Tzaneen and Modjadjiskloof, had a higher population density. In contrast, the Hoedspruit sub‐district had a scattered population due to a significant number of farms and game farms occupying large portions of land (Figure 5b). Greater Giyani sub‐district had the highest percentage (40%) of population living beyond 5 km from a PHC clinic, followed by Maruleng (28.5%), Greater Letaba (21.9%), Ba‐Phalaborwa (14.6%) and Greater Tzaneen (21.9%).

**FIGURE 6:**
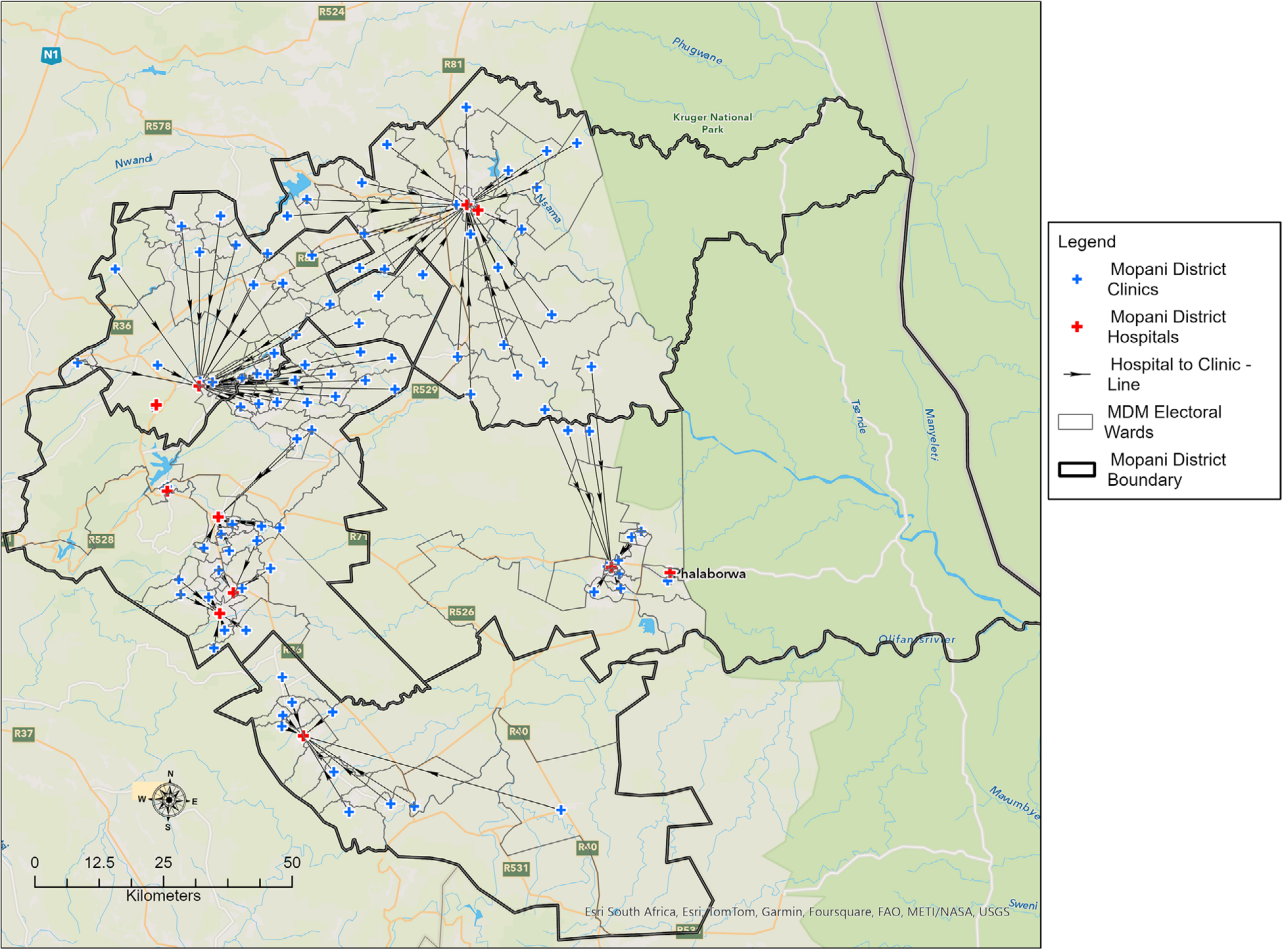
Distances from PHC clinics to the nearest district hospitals.

Figure 6 displays the distances between PHC clinics and the nearest district hospital. According to the data in Supporting Information 1, the average distance between PHC clinics and district hospitals in the Mopani District was 20.9 km (95%CI: 17.9–23.9 km). Out of the 105 PHC clinics, 34 (32.4%) were located within 0–10 km from the nearest district hospital, 19 (18.1%) were within 10–20 km, 19 (18.1%) were within 20–30 km and 33 (31.4%) were more than 30 km away.

On average, each clinic in the district served 11,546 people. The sub‐district of Ba‐Phalaborwa had the highest number of people per clinic, with 18,674 people per clinic. Greater Giyani had an average of 9490 people per clinic, Greater Letaba had 10,261, Greater Tzaneen had 12,342, and Maruleng had an average of 9365 people per clinic (Table 1).

Public transportation, primarily taxis, was vital for the district, with 86.5% of the population relying on it for mobility. Greater Tzaneen had 24 taxi facilities, of which only two in Tzaneen Town were formal. Ba‐Phalaborwa had 11 taxi facilities, with one in Phalaborwa Town being formal. Greater Letaba had 12 taxi facilities, with four being formal in Modjadjiskloof, Ga‐Kgapane, Sekgosese and Mokwakwaila. Greater Giyani had 18 taxi facilities, all of which were informal. Maruleng had six taxi facilities, with one being formal. Overall, 71 taxi facilities were present in the district, with only eight being formal and 63 informal (Table 2).

**TABLE 2:** Taxi facilities in Mopani District.

Sub‐district	Number of taxi facilities	Formal facilities	Informal facilities
Greater Tzaneen	24	2	22
Ba‐Phalaborwa	11	1	10
Greater Letaba	12	4	8
Greater Giyani	18	0	18
Maruleng	6	1	5
**Total**	**71**	**8**	**63**

The unemployment rate in Mopani District Municipality was 16.32%. Table 3 highlights the distribution of poverty within the Mopani District, delineated by sub‐districts and measured by the upper bound poverty line. In Ba‐Phalaborwa, 46.4% of the population, numbering 76,169 individuals, were living in poverty. Greater Giyani had the highest percentage of its population living in poverty, with 62.8, or 165,191 individuals. Greater Letaba reported 50.6% of its population, equivalent to 120,698 individuals, as living below the upper bound poverty line. In Greater Tzaneen, 49.1% of the population, or 211,783 individuals, were in poverty. Maruleng had 60.1% of its population, numbering 62,878 individuals, living in poverty. Overall, the Mopani District had 53.0% of its population, or 636,719 individuals, living below the upper bound poverty line, indicating significant socio‐economic challenges across the district.

**TABLE 3:** Distribution of population living below the upper bound poverty line in Mopani District by sub‐district.

Sub‐district	Population in poverty	Percentage of population in poverty
Ba‐Phalaborwa	76,169	46.4
Greater Giyani	165,191	62.8
Greater Letaba	120,698	50.6
Greater Tzaneen	211,783	49.1
Maruleng	62,878	60.1
Mopani District	636,719	53.0

## 4 Discussion

In this study, we employed GIS accessibility analysis to delineate the spatial distribution of COVID‐19 diagnostic services in the Mopani district, South Africa, shedding light on areas of both supply and demand within the healthcare system. With a total population of 1,202,916 in the Mopani district and 105 PHC clinics, an average service rate of approximately 11,456 individuals per clinic was found. Our findings underscored the equitable yet uneven distribution of PHC clinics across the district, with 78.5% of the population residing within 5 km of a clinic. Furthermore, 68.4% of these clinics were situated within a 30 km radius of a district hospital.

Ba‐Phalaborwa and Maruleng emerged as regions with good access to healthcare services, boasting high percentages of the population residing within 5 km of a clinic and a significant proportion of clinics located within 30 km of population centres. Similarly, Greater Letaba and Greater Tzaneen demonstrated relatively favourable accessibility metrics, with notable percentages of the population within close proximity to clinics and a substantial number of clinics within reasonable distances from population hubs. Conversely, Greater Giyani exhibited the lowest percentage of the population within 5 km of a clinic, indicating potential challenges in healthcare access. Additionally, only 53.6% of clinics in this district were located within 30 km of population centres, further complicating accessibility for certain individuals.

Our research adds to a growing body of evidence indicating that travel distance is a crucial factor affecting patient access to healthcare services [29, 30, 31]. In our study, the mean travelling distance to the closest PHC clinic is 2.95 km, which is encouraging compared to an urban study that reported an average travel distance of 3.5 km to access a healthcare facility [31]. However, other studies reported poor access to healthcare services among patients living in areas deprived of healthcare services, reporting a travel distance of more than 5 km to access healthcare services [32, 33, 34, 35]. Most of these studies reported that poor infrastructure, including transportation and utility systems, were some of the challenges to access healthcare services [33, 34, 35].

The district hospitals administered COVID‐19 polymerase chain reaction (PCR) testing, a method requiring 48–72 h for result processing [36, 37]. In contrast, PHC clinics offered COVID‐19 POC tests with immediate results, enabling rapid control measures. Despite the average travel distance from a clinic to the nearest hospital in the Mopani district being 20.9 km, deemed acceptable by South African standards, the delay in result transmission from district hospitals posed challenges for COVID‐19 control and management. Therefore, rapid testing for COVID‐19 provided at PHC facilities emerged as a viable alternative [38]. This may elucidate the difficulties encountered in tracking COVID‐19 in rural South Africa, where access to accurate diagnostic testing and the proximity between health clinics and district hospitals present logistical challenges.

Incorporating demographic and socio‐economic factors into our analysis provided valuable insights into healthcare accessibility dynamics within the Mopani District. Our study's findings aligned with existing literature, highlighting the interplay between population density, economic status and healthcare access [39, 40, 41]. The Mopani District presented a heterogeneous landscape characterized by densely populated urban centres juxtaposed with sparsely populated rural areas. Such demographic disparities can significantly influence healthcare accessibility, as urban centres typically have better infrastructure and a higher concentration of healthcare facilities compared to rural regions [42, 43, 44]. This dichotomy underscores the need for targeted interventions to bridge the healthcare gap between urban and rural communities.

Socio‐economic indicators further compound the accessibility challenges within the district. High unemployment rates and low‐income levels, particularly prevalent in rural areas, exacerbate the population's vulnerability and hinder their ability to access healthcare services [45, 46, 47]. Economic constraints can deter individuals from seeking timely medical care, leading to delayed diagnoses and exacerbation of health conditions [48, 49]. Addressing these socio‐economic barriers necessitates multifaceted approaches, including targeted financial assistance programmes and community‐based initiatives aimed at enhancing healthcare access and affordability [50, 51].

Moreover, limited public transport options emerge as a significant impediment to healthcare accessibility, particularly in rural areas [52, 53]. Reliance on walking or informal transport mechanisms imposes additional burdens on individuals seeking medical care, contributing to delays and disparities in healthcare access [54, 55]. Enhancing public transportation infrastructure and expanding affordable transport options are imperative steps towards mitigating these challenges and promoting equitable healthcare access across the district.

By integrating demographic and socio‐economic insights into our analysis, we gain a comprehensive understanding of the nuanced factors shaping healthcare accessibility within the Mopani District. Recognizing the complex interplay between population demographics, economic status and transport infrastructure is pivotal for devising targeted interventions aimed at narrowing healthcare disparities and fostering inclusive healthcare delivery systems in resource‐limited settings. Efforts to enhance healthcare access must be accompanied by broader socio‐economic development initiatives aimed at addressing underlying structural inequalities and empowering vulnerable populations to access essential healthcare services effectively.

Methodologically, our study employed network‐based travel distance as a proxy for accessibility, a pragmatic approach given available data. However, future research could strengthen such analyses by integrating friction surfaces, elevation data or empirical travel speed profiles, which allow for more realistic estimations of travel time and accessibility in heterogeneous rural landscapes [56, 57]. Recent work in other low‐ and middle‐income settings has shown the feasibility of these approaches, suggesting that combining distance‐based models with time‐based methods would enhance the precision and policy relevance of accessibility assessments in South Africa [58, 59, 60].

The findings of this study could assist policymakers in gaining a better understanding of the spatial distribution of COVID‐19 diagnostic services, facilitating the identification of areas lacking sufficient access to these services. Moreover, these insights can inform the development of integrated strategies aimed at optimizing accessibility and mitigating disparities, especially in resource‐limited settings. Additionally, the results of this study can serve as valuable guidance in decision‐making processes related to the planning of new healthcare facilities and the allocation of resources. However, to enhance the accuracy of accessibility assessments, future research endeavours should take into account factors such as transportation modes and geographical barriers, such as mountains and rivers, which can significantly influence travel distance and accessibility.

### 4.1 Strengths and Limitations

To the best of our knowledge, this study represents the first attempt to utilize geospatial analysis to ascertain the spatial distribution of COVID‐19 diagnostic services in this setting. The utilization of geospatial analyses has provided a robust platform for gaining insights into the accessibility of healthcare services, owing to the capability of processing large datasets quickly, efficiently and consistently [61]. Furthermore, these analyses possess adaptability and can be routinely updated, which is particularly advantageous in scenarios where the population undergoes growth or demographic changes.

Although our study has made substantial contributions to the comprehension of healthcare accessibility in rural areas, it is essential to acknowledge certain limitations. First, due to the lack of spatially precise residential data in the rural community, our study had to rely on catchment areas and PHC clinics as proxies for linking residential areas to the nearest district hospital. Additionally, our analysis predominantly utilized travel distance as a metric of accessibility, overlooking other critical factors such as cost, quality and cultural and linguistic barriers. A key limitation of this study is the reliance on travel distance rather than travel time. Travel time is often a more realistic proxy for patient access, as it incorporates road quality, terrain and transport options. Recent studies, such as those by Krishnakumari et al. [16] in Nepal and Kalonde et al. [17] in Malawi, demonstrate the advantages of time‐based modelling and validation against patient origin data. Incorporating such approaches in future studies would strengthen the empirical grounding and policy relevance of accessibility analyses in rural South Africa. Ensuring equitable access to healthcare services necessitates the consideration of these factors to accommodate the diverse needs of individuals and communities. Another limitation lies in the assumption that patients always opt for the nearest healthcare facility to their residence, whereas, in reality, patients in South Africa have the liberty to choose their healthcare provider. Furthermore, the well‐connected roads and public transport networks in the sub‐districts of rural South Africa facilitate access to healthcare facilities near workplaces or along transportation routes, which should be factored into future research endeavours.

### 4.2 Recommendations

On the basis of the findings of our study on the spatial distribution and accessibility of COVID‐19 diagnostic services in the Mopani District, we propose several recommendations aimed at addressing the identified challenges and enhancing healthcare accessibility. These recommendations are designed to optimize healthcare delivery and ensure equitable access to diagnostic services across the district.

- The study highlighted the significant role of public transport in healthcare accessibility, especially in rural areas. It is recommended to invest in improving public transportation infrastructure and expanding affordable transport options to reduce the reliance on walking or informal transport mechanisms. This will help mitigate the delays and disparities in healthcare access across the district.
- To overcome the spatial disparities in healthcare accessibility, particularly in rural areas, it is crucial to leverage technology. Implementing telemedicine, mobile health units and community outreach programmes can extend healthcare services to underserved populations. These measures will facilitate timely detection of diseases such as COVID‐19 and enhance overall healthcare delivery.
- The findings underscore the impact of socio‐economic factors on healthcare accessibility. Policymakers should integrate socio‐economic development initiatives with healthcare access programmes. This includes providing targeted financial assistance and community‐based initiatives to enhance healthcare affordability and access for vulnerable populations.
- The study identified areas with insufficient access to COVID‐19 diagnostic services. It is recommended to optimize the spatial distribution of diagnostic services by establishing new healthcare facilities in underserved areas and ensuring that existing facilities are well‐equipped to provide rapid testing services. This will help in managing and controlling the spread of infectious diseases more effectively.
- To maintain an up‐to‐date understanding of healthcare accessibility, it is essential to utilize geospatial analysis regularly. This approach can efficiently process large datasets and adapt to population growth or demographic changes, ensuring that healthcare services remain accessible to all communities.
- To create a more inclusive healthcare ecosystem, efforts should be made to address underlying structural inequalities. This involves empowering vulnerable populations through socio‐economic initiatives and ensuring that healthcare programmes are equitable and accessible to all individuals, regardless of their socio‐economic status.

## 5 Conclusion

In the Mopani District, the majority of the population (78.4%) resided within 5 km of COVID‐19 diagnostic services. However, significant challenges remain in terms of accessibility, particularly for timely disease diagnosis and healthcare service utilization. To address these issues, it is essential to enhance public transportation infrastructure, leverage technology and community‐based approaches, and implement targeted socio‐economic interventions. Improving the distribution of diagnostic services and conducting continuous geospatial analysis will further optimize healthcare accessibility. Addressing structural inequalities is also paramount to creating a more inclusive healthcare system. By prioritizing these actions, we can significantly improve healthcare access and outcomes for all residents in the Mopani District.

## Author Contributions

Kuhlula Maluleke conceptualized and wrote the draft manuscript under the supervision of Tivani Mashamba‐Thompson and Alfred Musekiwa. Kuhlula Maluleke and Ethel Baloyi collected and cleaned the data and wrote the results. David Mckelly analysed the data. Tivani Mashamba‐Thompson and Alfred Musekiwa critically reviewed and provided input to revise the manuscript. All authors have read and agreed to the published version of the manuscript.

## Funding

This work was supported by the National Research Foundation (MND210419595800) and Ninety‐One SA (Pty) Ltd (A1D350).

## Ethics Statement

This study was approved by the University of Pretoria Faculty of Health Research Ethics Committee (Reference No.: 655/2021, dated: 24 November 2021) and the Limpopo Department of Health ethics committee (Reference No.: LP_2021‐12‐007, dated: 27 February 2022). Data used in this study did not contain any personal identifiers.

## Consent

The authors have nothing to report.

## Conflicts of Interest

The authors declare no conflicts of interest.

## Supporting information



Supporting Information

## Data Availability

The datasets used and/or analysed during the current study are available from the corresponding author on reasonable request. The Limpopo‐DoH is the custodian for the DHIS data (available on request from Limpopo‐DoH: http://www.doh.limpopo.gov.za). The Mopani District Municipality is the custodian for the shapefile and all GIS‐based‐related data. The Mopani District population data are publicly available on the Statistics South Africa website (https://www.statssa.gov.za/). The geographic coordinates for the PHC clinic are publicly available on the NDoH website (https://www.health.gov.za/). Where possible, shapefiles or GIS workflows used in the analysis can be shared upon reasonable request to enhance replicability.

## References

[1] “Human Rights,” World Health Organization, accessed May 5, 2023, https://www.who.int/news‐room/fact‐sheets/detail/human‐rights‐and‐health.

[2] United Nations , “THE 17 GOALS,” Sustainable Development: United Nations, published 2021, https://sdgs.un.org/goals.

[3] K. Maluleke , A. Musekiwa , and T. Mashamba‐Thompson , “Evaluating Supply Chain Management of SARS‐CoV‐2 Point‐of‐Care (POC) Diagnostic Services in Primary Healthcare Clinics in Mopani District, Limpopo Province, South Africa,” PLoS ONE 18, no. 6 (2023): e0287477. 37368879 10.1371/journal.pone.0287477 PMC10298766

[4] “COVID‐19 Antigen Testing Guidelines 2020,” National Department of Health, accessed July 26, 2022, https://www.nicd.ac.za/wp‐content/uploads/2020/12/COVID‐19‐Antigen‐Testing‐Guidelines.pdf.

[5] “The Use of Antigen Testing for Diagnosis of SARS‐CoV‐2 in South Africa South Africa NICD,” National Institute for Communicable Diseases, published 2020, https://www.nicd.ac.za/wp‐content/uploads/2020/12/COVID‐19‐Antigen‐Testing‐Guidelines.pdf.

[6] “Diagnosis for All 2021,” Foundation for Innovative New Diagnostics, accessed July 26, 2022, https://www.finddx.org/.

[7] Z. M. McLaren , C. Ardington , and M. Leibbrandt , “Distance Decay and Persistent Health Care Disparities in South Africa,” BMC Health Services Research 14, no. 1 (2014): 541–548. 25367330 10.1186/s12913-014-0541-1 PMC4236491

[8] P. L. Delamater , J. P. Messina , A. M. Shortridge , and S. C. Grady , “Measuring Geographic Access to Health Care: Raster and Network‐Based Methods,” International Journal of Health Geographics 11, no. 1 (2012): 15. 22587023 10.1186/1476-072X-11-15 PMC3511293

[9] W. P. O'Meara , A. Noor , H. Gatakaa , B. Tsofa , F. E. McKenzie , and K. Marsh , “The Impact of Primary Health Care on Malaria Morbidity—Defining Access by Disease Burden,” Tropical Medicine & International Health 14, no. 1 (2009): 29–35. 19121148 10.1111/j.1365-3156.2008.02194.x PMC2658804

[10] A.‐L. Page , I. Ciglenecki , E. R. Jasmin , et al., “Geographic Distribution and Mortality Risk Factors During the Cholera Outbreak in a Rural Region of Haiti, 2010–2011,” PLOS Neglected Tropical Diseases 9, no. 3 (2015): e0003605. 25811860 10.1371/journal.pntd.0003605 PMC4374668

[11] D. J. Weiss , A. Nelson , C. A. Vargas‐Ruiz , et al., “Global Maps of Travel Time to Healthcare Facilities,” Nature Medicine 26, no. 12 (2020): 1835–1838. 10.1038/s41591-020-1059-1 32989313

[12] T. Kapwata , N. Morris , A. Campbell , et al., “Spatial Distribution of Extensively Drug‐Resistant Tuberculosis (XDR TB) Patients in KwaZulu‐Natal, South Africa,” PLoS ONE 12, no. 10 (2017): e0181797. 29028800 10.1371/journal.pone.0181797 PMC5640212

[13] F. Hierink , E. A. Okiro , A. Flahault , and N. Ray , “The Winding Road to Health: A Systematic Scoping Review on the Effect of Geographical Accessibility to Health Care on Infectious Diseases in Low‐ and Middle‐Income Countries,” PLoS ONE 16, no. 1 (2021): e0244921. 33395431 10.1371/journal.pone.0244921 PMC7781385

[14] J. L. Zelner , M. B. Murray , M. C. Becerra , et al., “Identifying Hotspots of Multidrug‐Resistant Tuberculosis Transmission Using Spatial and Molecular Genetic Data,” Journal of Infectious Diseases 213, no. 2 (2016): 287–894. 26175455 10.1093/infdis/jiv387 PMC4690150

[15] C. M. Smith , S. C. Le Comber , H. Fry , M. Bull , S. Leach , and A. C. Hayward , “Spatial Methods for Infectious Disease Outbreak Investigations: Systematic Literature Review,” Euro Surveillance 20, no. 39 (2015). 10.2807/1560-7917.ES.2015.20.39.30026 26536896

[16] P. K. Krishnakumari , H. Bakker , N. Lahrichi , et al., “Assessment of Geographical Accessibility to COVID‐19 Testing Facilities in Nepal (2021),” Lancet Regional Health—Southeast Asia 27 (2024): 100436. 39049977 10.1016/j.lansea.2024.100436 PMC11267066

[17] P. K. Kalonde , O. Tsoka , B. Chiepa , et al., “Mapping and Quantifying Travel Time to Define Health Facility Catchment Areas in Blantyre city in Malawi,” Communications Medicine 5, no. 1 (2025): 227. 40500310 10.1038/s43856-025-00845-3 PMC12159157

[18] T. Mabuka , N. Ncube , M. Ross , et al., “The Impact of Non‐Pharmaceutical Interventions on the First COVID‐19 Epidemic Wave in South Africa,” BMC Public Health [Electronic Resource] 23, no. 1 (2023): 1492. 37542267 10.1186/s12889-023-16162-0 PMC10403893

[19] T. G. Tshitangano , M. E. Setati , P. M. Mphekgwana , N. J. Ramalivhana , and S. F. Matlala , “Epidemiological Characteristics of COVID‐19 Inpatient Deaths During the First and Second Waves in Limpopo Province, South Africa,” Journal of Respiration 2, no. 2 (2022): 111–122.

[20] M. E. Sono‐Setati , P. M. Mphekgwana , L. N. Mabila , et al., eds., Health System‐ and Patient‐Related Factors Associated With COVID‐19 Mortality Among Hospitalized Patients in Limpopo Province of South Africa's Public Hospitals (Healthcare, 2022). 10.3390/healthcare10071338 PMC9323663 35885864

[21] P. Ellis , The Language of Research (Part 3): Cross Sectional Studies (Wounds UK, 2014), https://www.wounds‐uk.com/journals/issue/40/article‐details/the‐language‐of‐research‐part‐3‐cross‐sectional‐studies.

[22] “Provincial Profile Limpopo 2018,” Statistics South Africa, accessed July 26, 2022, https://www.statssa.gov.za/?p=11341.

[23] “Demographics,” Mopani District Municipality, published 2014, https://www.mopani.gov.za/docs/idp/REVIEWED%20%20IDP%202022‐2023%20FINAL.pdf.

[24] E. Baloyi , H. Mokgalaka , C. Green , and G. Mans , “Evaluating Public Ambulance Service Levels by Applying a GIS Based Accessibility Analysis Approach,” South African Journal of Geomatics 6, no. 2 (2017): 172–183.

[25] “PHC Facilities and Services,” National Department of Health, accessed July 26, 2022, https://www.health.gov.za/.

[26] M. Ngidi , G. Mans , D. McKelly , and Z. Sogoni , “Using a Hybrid Methodology of Dasyametric Mapping and Data Interpolation Techniques to Undertake Population Data (dis)Aggregation in South Africa,” South African Journal of Geomatics 6, no. 2 (2017): 232.

[27] “Dot Density ArcGIS Pro Documentation,” ESRI, published 2022, https://pro.arcgis.com/en/pro‐app/latest/help/mapping/layer‐properties/dot‐density.htm.

[28] “CSIR Guidelines for the Provision of Social Facilities in South Africa,” Council for Scientific and Industrial Research, published 2015, https://www.csir.co.za/sites/default/files/Documents/CSIR%20Guidelines_revised_reprintNov2015.pdf.

[29] Y. Lin , M. C. Wimberly , P. Da Rosa , J. Hoover , and W. F. Athas , “Geographic Access to Radiation Therapy Facilities and Disparities of Early‐Stage Breast Cancer Treatment,” Geospatial Health 13, no. 1 (2018): 622. 29772881 10.4081/gh.2018.622

[30] M. Charlton , J. Schlichting , C. Chioreso , M. Ward , and P. Vikas , “Challenges of Rural Cancer Care in the United States,” Oncology (Williston Park) 29, no. 9 (2015): 633–640. 26384798

[31] H. Mokgalaka , “Measuring Access to Primary Health Care: Use of a GIS‐Based Accessibility Analysis,” in Planning Africa 2014 Conference (International Convention Centre, 2014).

[32] S. Khairat , T. Haithcoat , S. Liu , et al., “Advancing Health Equity and Access Using Telemedicine: A Geospatial Assessment,” Journal of the American Medical Informatics Association 26, no. 8–9 (2019): 796–805. 31340022 10.1093/jamia/ocz108 PMC6696489

[33] R. H. M. Pereira , C. K. V. Braga , L. M. Servo , et al., “Geographic Access to COVID‐19 Healthcare in Brazil Using a Balanced Float Catchment Area Approach,” Social Science & Medicine 273 (2021): 113773. 33609968 10.1016/j.socscimed.2021.113773 PMC7879934

[34] A. Cattaneo , A. Nelson , and T. McMenomy , “Global Mapping of Urban–Rural Catchment Areas Reveals Unequal Access to Services,” Proceedings of the National Academy of Sciences of the United States of America 118, no. 2 (2021): e2011990118. 33431572 10.1073/pnas.2011990118 PMC7959575

[35] D. Kuupiel , K. M. Adu , F. Apiribu , et al., “Geographic Accessibility to Public Health Facilities Providing Tuberculosis Testing Services at Point‐of‐Care in the Upper East Region, Ghana,” BMC Public Health [Electronic Resource] 19, no. 1 (2019): 718. 31182068 10.1186/s12889-019-7052-2 PMC6558903

[36] C. Baxter , Q. Abdool Karim , and S. S. Abdool Karim , “Identifying SARS‐CoV‐2 Infections in South Africa: Balancing Public Health Imperatives With Saving Lives,” Biochemical and Biophysical Research Communications 538 (2021): 221–225. 33143875 10.1016/j.bbrc.2020.10.059 PMC7587129

[37] K. Maluleke , A. Musekiwa , S. Nxele , et al., “Co‐Creation of a Novel Approach for Improving Supply Chain Management for SARS‐CoV‐2 Point of Care Diagnostic Services in Mopani District, Limpopo Province: Nominal Group Technique,” Frontiers in Public Health 12 (2024): 1378508. 38784597 10.3389/fpubh.2024.1378508 PMC11111983

[38] K. Maluleke , A. Musekiwa , K. Kgarosi , et al., “A Scoping Review of Supply Chain Management Systems for Point of Care Diagnostic Services: Optimising COVID‐19 Testing Capacity in Resource‐Limited Settings,” Diagnostics 11, no. 12 (2021): 2299. 34943536 10.3390/diagnostics11122299 PMC8700402

[39] A. J. Comber , C. Brunsdon , and R. Radburn , “A Spatial Analysis of Variations in Health Access: Linking Geography, Socio‐Economic Status and Access Perceptions,” International Journal of Health Geographics 10 (2011): 1–11. 21787394 10.1186/1476-072X-10-44 PMC3155965

[40] C. A. Jones , T. S. Parker , M. C. Ahearn , A. K. Mishra , and J. N. Variyam , Health Status and Health Care Access of Farm and Rural Populations (USDA, 2009).

[41] Y.‐H. Ying , W.‐L. Lee , Y.‐C. Chi , M.‐J. Chen , and K. Chang , “Demographics, Socioeconomic Context, and the Spread of Infectious Disease: The Case of COVID‐19,” International Journal of Environmental Research and Public Health 19, no. 4 (2022): 2206. 35206390 10.3390/ijerph19042206 PMC8872250

[42] I. A. Ademiluyi and S. O. Aluko‐Arowolo , “Infrastructural Distribution of Healthcare Services in Nigeria: An Overview,” Journal of Geography and Regional Planning 2, no. 5 (2009): 104–110.

[43] A. Mustafa and C. Shekhar , “Is Quality and Availability of Facilities at Primary Health Centers (PHCs) Associated With Healthcare‐Seeking From PHCs in Rural India: An Exploratory Cross‐Sectional Analysis,” Clinical Epidemiology and Global Health 9 (2021): 293–298.

[44] S. Chatterjee and K. Sarkar , “Appraisal of Urban–Rural Disparities in Access to Health Care Facilities and Exposure to Health Risk Factors: A Case Study of Durgapur Industrial Region, India,” GeoJournal 87, no. 5 (2022): 4007–4024.

[45] Y. Sano and S. Mammen , “Mitigating the Impact of the Coronavirus Pandemic on Rural Low‐Income Families,” Journal of Family and Economic Issues 43, no. 2 (2022): 227–238. 35221641 10.1007/s10834-021-09800-5 PMC8860626

[46] G. C. Barron , G. Laryea‐Adjei , V. Vike‐Freiberga , et al., “Safeguarding People Living in Vulnerable Conditions in the COVID‐19 Era Through Universal Health Coverage and Social Protection,” Lancet Public Health 7, no. 1 (2022): e86–e92. 34906331 10.1016/S2468-2667(21)00235-8 PMC8665842

[47] T. Gashaw , B. Hagos , and M. Sisay , “Expected Impacts of COVID‐19: Considering Resource‐Limited Countries and Vulnerable Population,” Frontiers in Public Health 9 (2021): 614789. 34026704 10.3389/fpubh.2021.614789 PMC8131657

[48] M. A. Ezzat , “Identifying Barriers to Healthcare Access Among Underserved Populations: A Descriptive Study,” Journal of Advanced Analytics in Healthcare Management 7, no. 1 (2023): 1–17.

[49] V. Mogharab , M. Ostovar , J. Ruszkowski , et al., “Global Burden of the COVID‐19 Associated Patient‐Related Delay in Emergency Healthcare: A Panel of Systematic Review and Meta‐Analyses,” Globalization and Health 18, no. 1 (2022): 58. 35676714 10.1186/s12992-022-00836-2 PMC9175527

[50] A. Bekele , A. Alem , N. Seward , et al., “Barriers and Enablers to Improving Integrated Primary Healthcare for Non‐Communicable Diseases and Mental Health Conditions in Ethiopia: A Mixed Methods Study,” BMC Primary Care 25 (2024): 211. 38862874 10.1186/s12875-024-02458-6 PMC11167879

[51] E. Agyei and E. Kumah , “Navigating the Complex Terrain of Healthcare Systems in Sub‐Saharan Africa: Challenges and Opportunities for Progress,” Discover Health Systems 3, no. 1 (2024): 39.

[52] E. Vitale Brovarone and G. Cotella , “Improving Rural Accessibility: A Multilayer Approach,” Sustainability 12, no. 7 (2020): 2876.

[53] L. Chen , T. Chen , T. Lan , C. Chen , and J. Pan , “The Contributions of Population Distribution, Healthcare Resourcing, and Transportation Infrastructure to Spatial Accessibility of Health Care,” INQUIRY: The Journal of Health Care Organization, Provision, and Financing 60 (2023): 469580221146041. 10.1177/00469580221146041 PMC9837279 36629371

[54] S. Cooke , B. Ryseck , G. Siame , L. Molefe , A. Nkurunziza , and M. Zuidgeest . The Capabilities Approach to Vulnerable Non‐Motorised Transport Users in African Cities (Volvo Research and Educational Foundations Centre for Transport Studies University of Cape Town, 2021).

[55] J. S. Mindell and A. Curl , “Interactions Between Health and Travel Behaviour,” in Handbook of Travel Behaviour (Edward Elgar Publishing, 2024).

[56] Y. Liu , X. Cao , and T. Li , “Influence of Accessibility on Land Use and Landscape Pattern Based on Mapping Knowledge Domains: Review and Implications,” Journal of Advanced Transportation 2020, no. 1 (2020): 7985719.

[57] X. Gu , H. Wang , J. Lyu , M. Zhang , and Q. Ge , “Exploring the Impacts of Topography on Characteristics of Urban Road Network: A Global Empirical Analysis,” Cities 160 (2025): 105848.

[58] R. Chao , D. Xue , and B. Wang , “Evaluating Human Needs: A Study on the Spatial Justice of Medical Facility Services in Social Housing Communities in Guangzhou,” Land 13, no. 7 (2024): 1109.

[59] A. Cherono , Modelling Spatial Accessibility for Precision Targeting of School‐Based Interventions: A Case Study of Kilifi County (University of Nairobi, 2023).

[60] M. Tariverdi , M. Nunez‐Del‐Prado , N. Leonova , and J. Rentschler , “Measuring Accessibility to Public Services and Infrastructure Criticality for Disasters Risk Management,” Scientific Reports 13, no. 1 (2023): 1569. 36709371 10.1038/s41598-023-28460-z PMC9884248

[61] O. Lawal and F. E. Anyiam , “Modelling Geographic Accessibility to Primary Health Care Facilities: Combining Open Data and Geospatial Analysis,” Geo‐Spatial Information Science 22, no. 3 (2019): 174–184.

